# Sleep disordered breathing and cardiovascular risk in older patients initiating dialysis in the United States: a retrospective observational study using medicare data

**DOI:** 10.1186/s12882-016-0229-3

**Published:** 2016-02-09

**Authors:** C. Vaughan Tuohy, Maria E. Montez-Rath, Mintu Turakhia, Tara I. Chang, John W. Winkelman, Wolfgang C. Winkelmayer

**Affiliations:** Division of Nephrology, Department of Medicine, Stanford University School of Medicine, Palo Alto, CA USA; Division of Cardiovascular Medicine, Department of Medicine, Stanford University School of Medicine, Palo Alto, CA USA; Department of Health Research and Policy, Stanford University School of Medicine, Palo Alto, CA USA; Veterans Affairs Palo Alto Health Care System, Palo Alto, CA USA; Department of Psychiatry, Massachusetts General Hospital, Harvard Medical School, Boston, MA USA; Department of Medicine, New York University School of Medicine, New York, NY USA; Selzman Institute for Kidney Health, Section of Nephrology, Baylor College of Medicine, One Baylor Plaza, Suite ABBR R705, Houston, TX 77030 USA

**Keywords:** Obstructive sleep apnea, Autonomous neuropathy, End-stage renal disease, Risk assessment

## Abstract

**Background:**

Sleep disordered breathing (SDB) such as sleep apnea is associated with cardiovascular disease in the general population. However, little is known about the cardiovascular risks of SDB in patients with end-stage renal disease (ESRD).

**Methods:**

We identified Medicare fee-for-service beneficiaries aged ≥67 years initiating dialysis between 2004 and 2009. Outcomes of interest included all-cause mortality, incident myocardial infarction, ischemic stroke, and atrial fibrillation. We compared patients with and without diagnosed SDB using Cox proportional hazards regression.

**Results:**

Between 2004 and 2009, 184,217 older patients developed ESRD, of whom 15,121 (8.2 %) were previously diagnosed with SDB. Patients diagnosed with SDB were younger, more likely to be male and Caucasian, less Medicaid eligible, had more non-Nephrology clinic visits, higher body mass index, and more comorbidity. In analyses adjusting for demographics and BMI, diagnosed SDB was associated with higher risk of death and atrial fibrillation, but not associated with myocardial infarction or ischemic stroke risk. After further adjustment for all baseline characteristics, diagnosed SDB was associated with slightly lower risks of death (hazard ratio [HR]: 0.93, 95 % confidence interval [CI]: 0.91–0.96), myocardial infarction (HR: 0.92, CI: 0.87–0.98), and ischemic stroke (HR: 0.90, 95 % CI: 0.82–0.98), and not associated with atrial fibrillation (HR: 1.02, CI: 0.98–1.07).

**Conclusions:**

In older patients initiating dialysis in the U.S., diagnosed SDB was weakly associated with *lower* risks of death and important cardiovascular outcomes, thus adding to the list of established risk factors that are paradoxically associated with cardiovascular outcomes in the ESRD population.

## Background

Cardiovascular disease is the leading cause of death among patients with end-stage renal disease (ESRD), accounting for 38 % of deaths in the incident U.S. dialysis population between 2008 and 2010 [1]. Sleep-disordered breathing (SDB) encompasses a group of disorders characterized by abnormalities of respiratory pattern causing arousals and affecting the quantity of ventilation or arterial oxygen saturation during sleep [2], the most common being obstructive sleep apnea. It is increasingly recognized as a contributor to cardiovascular disease and has been shown to be independently associated with mortality [3–5] and cardiovascular events including stroke [3, 6–8], coronary artery disease [9–11], and cardiac arrhythmias including atrial fibrillation [12–16] in the general population. Treatment of SDB with continuous positive airway pressure (CPAP) has been shown to reduce systemic blood pressure in randomized studies [17] and reduce risk of cardiovascular events and death in observational studies [18–21], though evidence is conflicting [22].

It has been suggested that SDB is highly prevalent in patients with ESRD (>50 %) due to shared risk factors such as the metabolic syndrome [23, 24], and potentially some ESRD-specific factors including fluid shifts and overload, airway edema, uremia, and altered central and peripheral chemosensitivity [25–27].

Little is known about SDB and cardiovascular risk in patients with ESRD. We characterized the prevalence and features of diagnosed SDB in a large population of older patients initiating dialysis and hypothesized that diagnosed SDB was associated with all-cause mortality and the cardiovascular events of myocardial infarction (MI), ischemic stroke, and atrial fibrillation.

## Methods

### Study population

We used data from the United States Renal Data System (USRDS), and identified all patients over the age of 67 years who initiated maintenance dialysis between 2004 and 2009 and required that they had at least two years of continuous Medicare Parts A&B coverage at dialysis initiation. Patients with a history of MI, ischemic stroke, or atrial fibrillation in the two years preceding ESRD were excluded from the respective analyses of that outcome.

### Sleep disordered breathing

Patients diagnosed with SDB prior to initiation of maintenance dialysis were identified using International Classification of Diseases (9th Revision; ICD-9) diagnosis codes from Medicare billing claims during the two years preceding ESRD. We required a single inpatient code or two separate outpatient codes on different days to establish the diagnosis [28]. Prior to October 2005, 780.57 (sleep apnea not otherwise specified, NOS) was the sole ICD-9 code for SDB. In October 2005, several new ICD-9 codes for SDB were introduced. We defined SDB using codes 780.57, 327.2x (organic sleep apnea), 780.51 (insomnia with sleep apnea NOS) and 780.53 (hypersomnia with sleep apnea NOS) to capture patients diagnosed with SDB in both time periods. Because we found that very few (<5 %) patients received a diagnosis of SDB between 1996 and 2003 (Table 1), we limited our cohort to those initiating dialysis in the years 2004 through 2009. Given the changes in ICD-9 coding, we conducted a sensitivity analysis restricted to incident ESRD patients from 2007 through 2009 using only the more specific ICD-9 code 327.23 (obstructive sleep apnea; adult).

**Table 1:** Diagnosis of sleep disordered breathing and obstructive sleep apnea by year, 2004–2009

Year	Incident ESRD (N)	SDB (N)	SDB %	OSA (N)	OSA %
2004	31,741	1822	5.43	–	–
2005	31,553	2193	6.50	64	0.19
2006	29,715	2374	7.40	667	2.08
2007	27,401	2651	8.82	1243	4.14
2008	26,501	2864	9.75	1782	6.07
2009	25,753	3307	11.40	2462	8.47

Sleep disordered breathing (SDB) was defined as one inpatient or two separate outpatient Medicare billing claims with ICD-9 codes of “organic sleep apnea” (327.2x), “hypersomnia with sleep apnea NOS” (780.53), “insomnia with sleep apnea NOS” (780.51), or “sleep apnea NOS” (780.57)

Obstructive sleep apnea (OSA) was defined as one inpatient or two separate outpatient Medicare billing claims with ICD-9 codes of “obstructive sleep apnea (adult)” (327.23) only

Until October 2005, “sleep apnea NOS” (780.57) was the sole ICD-9 code for sleep disordered breathing, after which several new codes were added encompassing sleep apnea and related disorders

### Outcomes

The outcomes of interest were all-cause mortality and incident MI, ischemic stroke, and atrial fibrillation. Death was ascertained using the USRDS patient file. We defined MI by the presence of one inpatient claim with an ICD-9 primary diagnosis code of 410.xx or a secondary diagnosis code of 410.x1 or death from MI. Ischemic stroke was defined as one inpatient claim with a primary diagnosis code of 436, 437.1, 433.x1, or 434.x1 or death from stroke. Atrial fibrillation was defined as one inpatient or two outpatient billing claims with a diagnosis code of 427.3x.

For the outcome of death, follow-up was censored at end of study, December 31, 2009. For the outcomes of MI, stroke, and atrial fibrillation, follow-up was censored at end of study, loss of Medicare fee-for-service coverage, or death. We did not censor at kidney transplantation, which was a rare occurrence (~1.1 % of patients) and similar between exposure groups.

### Patient characteristics

From the USRDS patient file, we gathered demographic information including age, sex, race (white; black; other), Hispanic ethnicity, and cause of ESRD. Presence of a large number of pre-existing comorbidities was ascertained using specific ICD-9 codes from the two years preceding ESRD by at least one inpatient or two outpatient claims on different days. We ascertained dual Medicaid-Medicare eligibility, serum albumin, and body mass index (BMI) at initiation of maintenance dialysis from the Medical Evidence Report (form CMS-2728). Estimated glomerular filtration rate was provided in the USRDS based on reported serum creatinine concentrations. We also quantified health care utilization in the year prior to initiation of dialysis by determining whether the patient was in a skilled nursing facility, the number of days spent in hospital, and the number of non-nephrology outpatient visits.

### Statistical analysis

Our cohort was divided into patients with versus without diagnosed SDB. Baseline categorical variables were expressed as percentages and continuous variables as medians and interquartile range. Unadjusted incidence rates, defined as the number of events over person-time observed, were calculated for each outcome.

Time from incident ESRD to each outcome of interest was analyzed using the Kaplan-Meier method and Cox proportional hazards regression. We estimated hazard ratios (HR) and their corresponding 95 % confidence intervals (CI) using four nested Cox models for each outcome: 1) unadjusted; 2) adjusted for age, sex, race, Hispanic ethnicity, and dual Medicaid-Medicare eligibility; 3) additionally adjusted for BMI (including BMI squared); 4) additionally adjusted for all covariates listed in Table 2. Since we removed patients with a history of the outcomes of interest, the corresponding covariate was not included in the final model. All models were stratified by incident year. We used log (−log) survival curves and plots of Loess-smoothed scaled Schoenfeld residuals to assess the adequacy of the Cox proportional hazards models.

**Table 2:** Baseline characteristics and recorded diagnoses of older individuals initiating dialysis, by presence of sleep disordered breathing

	All	SDB-	SDB+
	*N* = 184,217	*N* = 169,096	*N* = 15,121
Patient demographics			
Patient age	77 (72–82)	77 (72–82)	74 (70–79)
Female	46.8	47.6	37.7
Race			
White	77.6	77.2	82.3
Black	18.6	18.8	16.0
Other	3.8	3.9	1.7
Hispanic ethnicity	7.4	7.6	5.6
Dual Medicare-Medicaid Eligibility	22.0	22.3	18.5
Cause of end-stage renal disease			
Diabetes	39.4	38.3	51.6
Hypertension	36.8	37.4	29.0
Glomerulonephritis	5.4	5.4	4.4
Other	17.6	17.9	14.4
Missing	0.9	0.9	0.6
Dialysis modality			
Hemodialysis	94.9	94.9	95.1
Peritoneal Dialysis	4.6	4.6	4.6
Missing	0.5	0.5	0.2
Skilled Nursing Facility Utilization	15.0	15.0	15.4
Hospital days (N)	5 (0–17)	5 (0–16)	6 (0–21)
Non-nephrology clinic visits (N)	19 (4–35)	18 (4–34)	28 (3–49)
Body Mass Index (BMI) (kg/m^2^)	26.0 (22.7–30.5)	25.7 (22.5–29.9)	31.4 (26.7–37.0)
BMI % missing data	2.0	2.0	1.8
Comorbidities			
Obesity	9.5	7.4	32.5
Diabetes	62.4	60.9	78.4
Dyslipidemia	67.6	66.6	79.1
Systemic hypertension	96.9	96.7	99.1
Pulmonary hypertension	10.4	9.2	23.8
Heart failure	67.7	66.4	82.8
Valvular disease	39.0	38.3	47.2
Myocardial infarction	15.9	15.8	17.2
Unstable angina	20.7	20.3	25.3
Coronary artery bypass graft	7.7	7.5	9.9
Percutaneous coronary intervention	5.4	5.3	7.0
Peripheral vascular disease	38.3	37.9	42.6
Cerebrovascular disease	26.4	25.2	28.8
Ischemic stroke	16.8	16.7	18.0
Transient ischemic attack	7.8	7.7	8.8
Central nervous system bleed	1.5	1.5	1.8
Atrial fibrillation	32.1	31.2	42.8
Ventricular fibrillation	8.5	8.1	13.2
Other cardiac arrhythmias	21.7	21.2	27.3
Pacemaker	4.6	4.5	6.4
Implantable cardiac defibrillator	2.4	2.2	4.2
Hypothyroidism	22.0	21.6	26.3
Hyperparathyroidism	5.5	5.5	6.2
Cancer	21.6	21.7	20.9
Liver disease	7.5	7.4	8.3
Lung disease	42.4	40.4	64.8
Rheumatologic disease	5.7	5.6	6.9
Gastrointestinal Bleed	21.5	21.2	24.5
Peptic ulcer disease	4.1	4.1	4.2
Human Immunodeficiency Virus	0.1	0.1	0.1
Paralysis	3.1	3.1	3.2
Depression	12.9	12.5	17.9
Dementia	10.5	10.5	10.2
Psychosis	4.9	4.8	5.7
Alcohol abuse	1.7	1.7	1.4
Tobacco abuse	5.9	6.0	5.0
Drug abuse	1.2	1.2	1.7
Acidosis	22.8	23.0	20.8
Alkalosis	1.8	1.7	3.9
Mixed acid–base disorder	1.4	1.3	2.5
Hyponatremia	14.1	13.9	16.1
Hypernatremia	3.6	3.5	4.5
Hypokalemia	14.0	13.6	18.2
Hyperkalemia	31.9	31.7	34.1
Hypocalcemia	3.3	3.4	2.5
Hypercalcemia	2.9	2.9	3.1
Disorders of phosphorus metabolism	4.0	4.0	3.9
Disorders of magnesium metabolism	3.2	3.1	3.7
Laboratory measurements			
Albumin, g/dL	3.2 (2.7–3.6)	3.2 (2.7–3.6)	3.2 (2.8–3.6)
Albumin % missing data	24.4	24.3	25.8
Hemoglobin, g/dL	10.2 (9.2–11.2)	10.2 (9.2–11.2)	10.2 (9.3–11.3)
Hemoglobin % missing data	8.8	8.8	9.4
Estimated glomerular filtration rate (eGFR), mL/min/1.73 m^2^	10.7 (8.0–14.2)	10.6 (7.9–14.1)	12.0 (9.1–15.9)
eGFR % missing data	2.7	2.6	3.7

*SDB* sleep disordered breathing

About 29 % of patients had at least one variable missing, with the proportion of variables with data missing ranging from less than 0.5 % (dialysis modality) to 25 % (albumin; Table 2). We assumed the data to be missing at random and used multiple imputation (using SAS proc mi) through the joint modeling approach to obtain 7 imputed datasets [29]. One dataset was used for model checking and to the remaining 6 we fitted the stratified Cox proportional hazard models. The results of the 6 fitted models were then combined using the rules described by Little and Rubin (using SAS proc mianalyze) [30]. Imputation was done separately for each outcome analyzed and the imputation model included all variables used in the analyses. Height or weight was used to impute BMI if only one was available. Complete case analyses, a less preferred option, were also conducted and yielded quantitatively similar results albeit with wider confidence limits owing to the reduced sample size (results not shown).

This study was approved by an institutional review board at Stanford University. All analyses were conducted using SAS software, version 9.3 (www.sas.com) and R (www.r-project.org).

## Results

We identified 184,217 patients who developed ESRD between 2004 and 2009 and met inclusion criteria for our primary cohort. Of these, 15,121 (8.2 %) were diagnosed with SDB. Diagnosis of SDB increased steadily throughout the study period, from 5.4 % in 2004 to 11.4 % in 2009 (Table 1). Patients with diagnosed SDB (SDB+) were slightly younger, more often male and Caucasian, and had higher prevalence of a wide range of pre-existing comorbidities as compared to patients without diagnosed SDB (SDB-; Table 2).

### Mortality

**Fig. 1:**
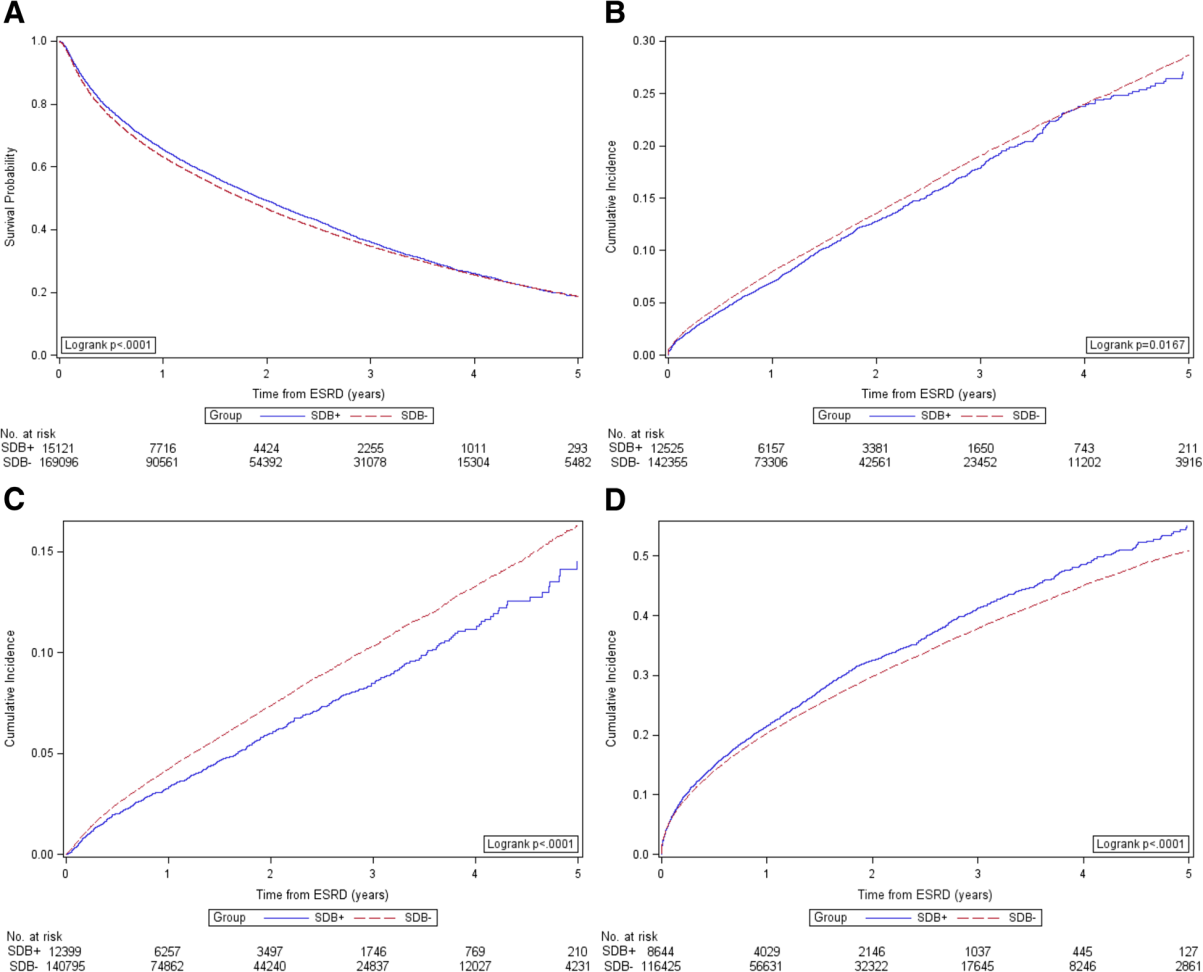
Kaplan-Meier Product Limit Estimates of Study Outcomes. Panel **a** Patient Survival. Panel **b** Myocardial Infarction. Panel **c** Ischemic Stroke. Panel **d** Atrial Fibrillation

**Fig. 2:**
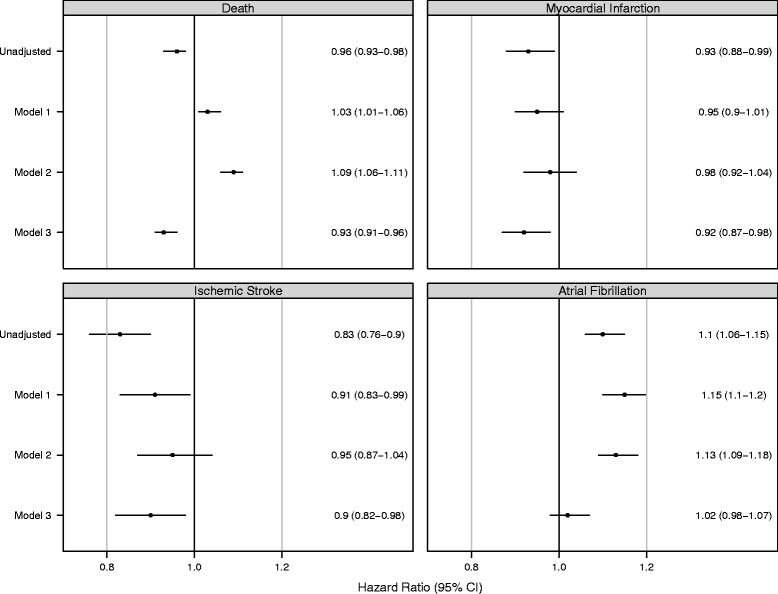
Unadjusted and Adjusted Hazard Ratios for Study Outcomes. Stratified Cox analysis with multiple imputation to handle missing data. All models stratified by Year of dialysis initiation. Model 1 is adjusted for age, sex, race, Hispanic ethnicity, and Medicaid eligibility. Model 2 is additionally adjusted for BMI. Model 3 is additionally adjusted for all covariates in Table 1. Ischemic stroke includes events related to death from any kind of stroke. Myocardial infarction includes death from myocardial infarction

During a mean follow up of 1.60 years (range 0–6 years), 8248 (54.6 %) SDB+ patients and 103,731 (61.3 %) SDB- patients died (Table 3; Fig. 1a). SDB diagnosis was associated with a slight survival advantage, which dissipated over time (Fig. 1a). The unadjusted hazard ratio (HR) for death was 0.96 (95 % CI: 0.93–0.98) in the SDB+ vs. the SDB- group (Fig. 2). When adjusting for demographic characteristics (Model 1) and for BMI (Model 2), SDB+ patients had higher mortality (Model 2 HR 1.09, 95 % CI: 1.06–1.11). However, after adjusting for all additional baseline characteristics (Model 3), the SDB+ group had lower associated mortality (HR: 0.93, 95 % CI: 0.91–0.96) compared with the SDB- group.

**Table 3:** Follow-up and study outcomes

Outcome	SDB-	SDB+
Death	*N* = 169,096	*N* = 15,121
Number of events	103,731	8248
Person-time at risk (years)	271,789	22,434
Incidence rate (events/100 person-years)	38.2	36.8
Myocardial infarction (non-fatal or fatal)	*N* = 142,355	*N* = 12,525
Number of events	16,217	1241
Person-time at risk (years)	216,722	17,639
Incidence rate (events/100 person-years)	7.48	7.04
Ischemic stroke (non-fatal or fatal)	*N* = 140,795	*N* = 12,399
Number of events	8604	576
Person-time at risk (years)	222,419	17,962
Incidence rate (events/100 person-years)	3.87	3.21
Incident atrial fibrillation	*N* = 116,425	*N* = 8644
Number of events	30,745	2369
Person-time at risk (years)	166,609	11,416
Incidence rate (events/100 person-years)	18.5	20.8

### Cardiovascular outcomes

Excluding patients who had an MI in the two years preceding ESRD, 1241 (9.9 %) of SDB+ patients and 16,217 (11.4 %) of SDB- patients had a fatal or non-fatal MI during the study period (Table 3; Fig. 1b). We found a lower risk for MI in the SDB+ group vs. the SDB- group in unadjusted and adjusted models (Fig. 2).

Excluding patients who had an ischemic stroke in the two years preceding ESRD, 576 (4.7 %) of SDB+ patients and 8604 (5.6 %) of SDB- patients had a fatal or non-fatal ischemic stroke during the study period (Table 3; Fig. 1c). Similar to the results for MI, we found a lower risk of ischemic stroke in the SDB+ group versus the SDB- group (Fig. 2).

Excluding patients who had diagnosed atrial fibrillation in the two years preceding ESRD, 2369 (27.4 %) of SDB+ patients and 30,745 (26.4 %) of SDB- patients developed new atrial fibrillation during follow up (Table 3; Fig. 1d). In contrast the results for MI and ischemic stroke, SDB was associated with higher incidence of newly diagnosed atrial fibrillation in unadjusted models (HR 1.10; 95 % CI: 1.06–1.15; Fig. 2). However, the results were no longer statistically significant in fully adjusted analyses (Model 3 HR: 1.02, 95 % CI: 0.98–1.07; Fig. 2).

### Sensitivity analyses

**Fig. 3:**
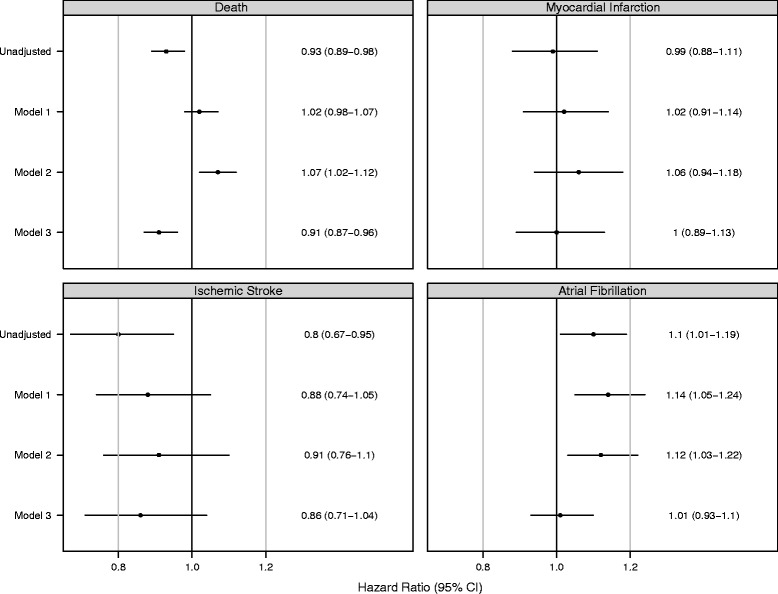
Sensitivity Analysis: Unadjusted and Adjusted Hazard Ratios for Study Outcomes using Obstructive Sleep Apnea Definition, 2007–2009. Stratified Cox analysis with multiple imputation to handle missing data. All models stratified by Year of dialysis initiation. Model 1 is adjusted for age, sex, race, Hispanic ethnicity, and Medicaid eligibility. Model 2 is additionally adjusted for BMI. Model 3 is additionally adjusted for all covariates in Table 1. Ischemic stroke includes events related to death from any kind of stroke. Myocardial infarction includes death from myocardial infarction

In sensitivity analyses using only the more specific obstructive sleep apnea (OSA) diagnosis code, we found that of 81,538 patients, 5448 (6.3 %) had a diagnosis code for OSA. The point estimates of the hazard ratios for death, stroke, and atrial fibrillation were similar using the OSA definition as compared to the SDB definition (Fig. 3). However, given the smaller sample sizes, the confidence intervals were wider, and some results were no longer statistically significant. The point estimates of the hazard ratios for MI in the SDB+ vs. the SDB- group were slightly higher in the sensitivity analysis than in the primary analysis and non-significant for all four models.

Sensitivity analyses using a competing risks approach did not materially change our results (data not shown).

## Discussion

In this large population of older patients initiating dialysis, diagnosis of SDB in the two years prior to ESRD was associated with slightly elevated risks of death and atrial fibrillation, and no difference in risks of MI or ischemic stroke when controlling for demographic factors and BMI (Model 2). However, after additional adjustment for a large number of pre-existing comorbid conditions, laboratory values, cause of ESRD, dialysis modality, and several health care utilization variables (Model 3), SDB was associated with lower risks of death, MI, and ischemic stroke, while no association was found with incident atrial fibrillation. A sensitivity analysis using the more specific diagnosis of OSA yielded similar results. Overall, this study does not provide evidence supporting a strong association of SDB with mortality or cardiovascular outcomes in older, incident dialysis patients.

Our findings were in contrast with several studies conducted in the general population, which suggested that SDB was a strong and treatable risk factor for cardiovascular events. In a prospective cohort of men and women over 50 years old from outpatient sleep clinics [3, 10], severe OSA was associated with incident coronary events and cardiovascular death (HR 2.82, CI 1.46–5.45). In the same study, mild OSA was associated with stroke or transient ischemic attack (relative risk [RR] 3.02; CI 1.27–7.21), but not with mortality (HR 1.70, CI 0.92–3.16). In the Sleep Heart Health Study [8], a large U.S. multi-center community-based prospective cohort of patients over 40 years old, moderate to severe OSA was associated with increased risk for incident stroke in men (HR 2.86, CI 1.10–7.39), but was not associated with incident MI, death from MI, or need for revascularization [9] . However, the mean age in the Sleep Heart Health Study was substantially younger than in our cohort. A Spanish study of community-dwelling elderly patients over 70 years of age did find an increased risk of ischemic stroke with severe OSA (HR 2.52, CI 1.04–6.10), but this analysis did not control for important potential confounders such as comorbid conditions or BMI.^7^

Multiple studies in the general population have found an association between SDB and atrial fibrillation [12–15]. SDB has also been associated with recurrent atrial fibrillation after ablation [31], and apneic events have been shown to directly trigger nocturnal arrhythmias [16]. Our findings in patients with ESRD are largely consistent, though the association we found was fairly weak. As with MI and stroke, this could partly reflect age differences. In a study of patients without baseline atrial fibrillation undergoing polysomnography at the Mayo Clinic in Minnesota, mild or worse OSA (AHI > 5) was associated with incident atrial fibrillation in patients under 65, but not in patients over 65 [13]. In the Sleep Heart Health Study, SDB was associated with nocturnal arrhythmias, but there was attenuation of risk with increasing age [14]. More recently, a study of community-dwelling men over 65 years of age (Outcomes of Sleep Disorders in Older Men Study) did find an independent association between severity of SDB and incident atrial fibrillation [15]. In particular, central sleep apnea (CSA) was more strongly associated with atrial fibrillation, whereas OSA was more closely associated with complex ventricular ectopy. Given that CSA may be more prevalent in ESRD, a stronger association between SDB and atrial fibrillation might have been expected, but this was not the case.

A few small studies have already been conducted on SDB and cardiovascular events in ESRD. In a cohort (*n* = 50) of Italian patients with ESRD, an independent association was found between nocturnal hypoxemia and cardiovascular events [32]. Recently, two small studies of patients undergoing peritoneal dialysis in Taiwan (*n* = 93) and hemodialysis in Japan (*n* = 94) found significant independent associations between SDB and cardiovascular events [33, 34]. These studies differ from ours in that they were conducted in small groups of selected patients attending sleep clinics and assessed a broader range of cardiovascular outcomes (including heart failure, angina, other arrhythmias) as a combined outcome. They also used polysomnography to screen for SDB, an advantage over our study design. In contrast to their findings, our study suggests that while SDB may be a contributor to incident atrial fibrillation, it is probably not a significant factor driving cardiovascular events and mortality in patients with ESRD.

There are several reasons why SDB might be a less powerful risk factor for cardiovascular events in ESRD. Fluid shifts, heart failure, fluid overload, uremia, and other ESRD-specific factors may contribute to the pathophysiology of SDB and differentially mediate cardiovascular risk. Indeed, prior research suggests that risk factors for SDB differ in patients with ESRD, and that SDB may present somewhat differently [35]. In the dialysis population, SDB may be more frequently central rather than obstructive [24], and these patients may present with less “Pickwickian” features [35]. There are also strong competing risks for cardiovascular events in ESRD, which could attenuate the effect of SDB. It is also relevant that comorbidities associated with SDB (e.g., diabetes and the metabolic syndrome) probably contribute to the development of ESRD in the first place. It has even been suggested that SDB might independently contribute to chronic kidney disease [36], but once ESRD is reached, SDB might become less important. That is, if patients with SDB are more likely to die before developing ESRD, then those patients initiating dialysis constitute the “surviving fittest”. There are several examples of established risk factors for cardiovascular disease that are paradoxically protective in ESRD, such as obesity and higher blood pressure, which are associated with longer survival in patients on dialysis [37–39]. Similar explanations may be relevant to SDB in ESRD.

Our study has a few important limitations. First, we used Medicare claims diagnosis codes to identify patient with SDB rather than polysomnography, the diagnostic gold standard. Estimates of the prevalence of sleep apnea in the ESRD population using polysomnography range from 30 to 80 % [24, 40], although the prevalence of SDB in older patients with ESRD has not been established. SDB is generally underrecognized, and it may be further underdiagnosed in patients with ESRD due to attention placed on other health concerns or because of the different characteristics of SDB in this population. The prevalence of SDB was only ~8 % in our study, reflecting underdiagnosis. We did not have clinical information on polysomnography or specific treatments for SDB in this study. There is potential for bias if patients diagnosed with SDB were systematically different from those with occult SDB. Patients with ESRD often have a high burden of comorbid illness, can be physically limited, and carry a poor prognosis, so perhaps healthier patients with SDB were more likely undergo work-up. However, it is plausible to consider that patients diagnosed with SDB were the most severe cases, which makes the lack, or paradoxical direction, of the associations found even more striking. Several baseline factors may have been causes, some consequences, and again some both causes and consequences of the exposure of interest. Owing to the timeline of available data, we were unable to distinguish among these categories. Hence, the true associations are likely to be bound by the respective associations in models 1 and 3. Another potential confounder may be differences in access to care, with better access leading to higher probability of SDB diagnosis and thus better outcomes. However, at the very least all patients in this study had continuous insurance coverage for at least 2 years prior to ESRD through the federal Medicare program. Our study did not include patients under 67 years of age, in younger patients we would not have pre-ESRD Medicare claims for study (the age threshold for Medicare eligibility is 65 years), so our findings may not be generalizable to younger individuals with ESRD. Finally, follow-up was relatively short in this study mostly owing to the very high mortality rate (~38 deaths per 100 person-years) in this population of older patients with ESRD.

Our study has several strengths, especially its national scope and broad generalizability to the older U.S. dialysis population. Patients with and without diagnosed SDB were also well characterized on a wide range of demographic characteristics, comorbid conditions, and laboratory variables, using data from several sources. We found that SDB diagnosis was associated with higher prevalence of several baseline comorbid diagnoses including obesity, diabetes, hyperlipidemia, depression, cardiovascular disease, heart failure, arrhythmias, and pulmonary hypertension, all of which have been associated with SDB in the general population.

## Conclusions

In summary, in demographics and BMI-adjusted analyses physician-diagnosed SDB was associated with slightly increased risks of mortality and atrial fibrillation, and no difference in the risks of myocardial infarction or ischemic stroke in older patients initiating dialysis. Adjustment for a large number of comorbidities and other parameters yielded protective associations between SDB and most cardiovascular outcomes. Though we cannot exclude the possibility that SDB diagnosed by polysomnography would carry different cardiovascular risks, our study suggests that SDB may not be as important a risk factor for cardiovascular events or atrial fibrillation in older patients with ESRD as in the general population. Further studies are needed to explain our findings, especially ones that prospectively screen patients for SDB using validated screening methods (questionnaires) or accepted testing methods. Studies are also needed to assess the effect of treatments for SDB such as CPAP and alternative dialysis modalities on cardiovascular outcomes. With improved understanding of this potential risk factor for cardiovascular disease, we can better inform the screening and treatment decisions of providers caring for patients living with ESRD.

## Abbreviations

BMI: body mass index
CI: confidence interval
CMS: Centers for Medicare and Medicaid Services
ESRD: end-stage renal disease
HR: hazard ratio
ICD-9: international classification of diseases, 9^th^ revision
MI: myocardial infarction
NOS: not otherwise specified
OSA: obstructive sleep apnea
SDB: sleep disordered breathing
USRDS: United States Renal Data System
